# A Mixed-Methods Examination of Interdisciplinary Strategies for Addressing Trauma and Chronic Pain in Group Therapy

**DOI:** 10.3390/healthcare14121622

**Published:** 2026-06-09

**Authors:** Kara M. Schneider, Dodie Limberg, Krista M. Schneider, Claire Balane, Jessica Barnes, Brittany Sandonato, Ashley J. Blount

**Affiliations:** 1 Counseling Department, University of Nebraska at Omaha, Omaha, NE 68182, USA; ablount@unomaha.edu; 2 Department of Educational and Developmental Science, University of South Carolina, Columbia, SC 29208, USA; dlimberg@sc.edu (D.L.); jmb25@email.sc.edu (J.B.); sandonab@email.sc.edu (B.S.); 3 Independent Researcher, Omaha, NE 68114, USA; krschneid03@gmail.com (K.M.S.); claire@ccaomaha.com (C.B.)

**Keywords:** chronic pain, trauma, PTSD, chronic stress, group counseling, interdisciplinary care, physical therapy

## Abstract

**Highlights:**

- The interdisciplinary group intervention was feasible and acceptable, with high attendance and fidelity.
- Pre/post outcomes suggested improvements in trauma symptoms, fear of movement, pain, and postural neutrality, though findings are preliminary given the small sample.
- Interdisciplinary, mind–body group interventions may provide a feasible model for integrating physical and mental health care for clients with co-occurring trauma and chronic pain.
- Findings highlight the value of cross-disciplinary training and collaboration, suggesting that counselor–physical therapist partnerships may expand the scope and impact of trauma-informed care in community settings.

**Abstract:**

**Background/Objectives:** Trauma and chronic pain frequently co-occur and mutually reinforce functional impairment, yet few counseling interventions integrate somatic and psychological approaches. This study had two primary objectives: (1) to evaluate the feasibility and acceptability of an interdisciplinary trauma–chronic pain group intervention, and (2) to explore preliminary clinical outcomes related to trauma symptoms, fear of movement, and pain through an interdisciplinary group intervention combining trauma-informed counseling with physical therapy grounded in Postural Restoration Institute (PRI) principles. **Methods:** A convergent mixed-methods design was used, wherein quantitative and qualitative data were collected concurrently, analyzed separately, and integrated during interpretation to provide a comprehensive understanding of intervention outcomes. Fifteen adults with chronic pain (≥3 months) and clinically significant trauma symptoms (PCL-5 ≥ 31) completed a six-week, 90 min group program co-facilitated by a counselor and a physical therapist. Quantitative measures included weekly pain (BPI), pre/post trauma symptoms (PCL-5), fear of movement (TSK), group climate (GCQ), and postural neutrality. Qualitative data included weekly journals, photographs, open-ended post-surveys, and focus groups, analyzed using inductive thematic analysis. **Results:** Participants attended most sessions (96.7% overall attendance) with no dropouts and high fidelity (mean 2.89/3). Pre/post analyses indicated statistically significant within-group reductions in trauma symptoms and fear of movement and improvements in pain and postural neutrality; however, findings should be interpreted as preliminary given the pilot design and small sample size. Qualitative themes highlighted the persistent burden of pain/trauma, increased mind–body awareness, emotional regulation, and validation through group support. **Conclusions:** An interdisciplinary, PRI-informed, trauma-informed group model was obtained. This model demonstrates preliminary feasibility and acceptability and suggests potential benefit; however, findings are derived from a small, non-controlled pilot study and require further validation. Future research should employ larger, controlled, and longitudinal designs.

## 1. Introduction

Trauma is a pervasive public health concern associated with significant psychological, physical, and functional impairment across the lifespan [1,2]. Trauma has been defined as experiences that are physically or emotionally harmful and that have lasting adverse effects on individuals’ well-being and functioning [3]. Exposure to traumatic events is common, with estimates suggesting that approximately 70% of individuals globally will experience at least one traumatic event during their lifetime [1]. Trauma is associated with a range of mental health concerns, including posttraumatic stress symptoms, emotional dysregulation, and interpersonal difficulties, even when individuals do not meet full diagnostic criteria for posttraumatic stress disorder [4,5].

Chronic pain is similarly prevalent and debilitating, affecting approximately one in five adults in the United States [6,7]. Globally, chronic pain remains a leading contributor to disability, affecting populations across diverse healthcare systems [8]. Chronic pain is defined as pain persisting for three months or longer and is characterized not only by sensory discomfort but also by emotional and cognitive distress [9]. Individuals with chronic pain frequently experience reduced quality of life, functional limitations, and elevated rates of depression and anxiety [9,10]. These psychological and physical challenges often co-occur, highlighting the need for treatment approaches that address both domains simultaneously. This burden is not evenly distributed, with individuals in low- and middle-income countries disproportionately affected due to limited access to comprehensive pain management and rehabilitation services [8].

The intersection of trauma and chronic pain is particularly important for counselors, as these conditions frequently co-occur and mutually reinforce one another. Individuals with chronic pain often report histories of trauma and PTSD symptoms, and the presence of trauma has been associated with increased pain severity, poorer sleep, and greater functional impairment [11,12,13,14]. Trauma-related alterations in stress and threat processing may contribute to heightened pain sensitivity through mechanisms such as central sensitization, in which the nervous system becomes hyper-responsive following traumatic stress [13,14]. As a result, psychological distress and physical pain may operate in a bidirectional cycle that maintains both conditions over time. Longitudinal and prospective research suggests that trauma exposure predicts the onset, severity, and persistence of chronic pain over time, supporting a bidirectional relationship between posttraumatic stress and pain processes [15,16,17,18].

Neurobiological research further supports the interconnected nature of trauma and pain. Shared neural pathways implicated in threat perception, emotional regulation, and pain processing suggest overlapping neurocircuitry underlying both trauma-related distress and chronic pain experiences [19,20]. Trauma-related physiological responses, including heightened arousal and stress reactivity, may contribute to hyperalgesia and the persistence of pain symptoms [21,22]. These findings underscore the importance of treatment approaches that address both emotional and somatic processes in an integrated manner. These shared neurobiological pathways informed the intervention design, particularly the integration of somatic regulation strategies aimed at modulating autonomic arousal and reducing threat sensitivity. Despite growing support for interdisciplinary approaches, limited research has examined structured, manualized interventions integrating physical therapy and trauma-informed counseling within group settings, particularly using mixed-methods designs to evaluate both clinical outcomes and participant experience.

### 1.1. The Need for Interdisciplinary Care

Given the high prevalence of trauma among clients seeking mental health services, counselors are increasingly expected to assess and treat trauma-related symptoms that intersect with physical health concerns [1,23]. Trauma-informed care frameworks emphasize that all counselors, not only trauma specialists, must understand how trauma affects both psychological and physiological functioning in order to provide effective and ethically responsive care [24,25].

Interdisciplinary care models grounded in the biopsychosocial perspective offer a promising avenue for addressing the complex and reciprocal relationship between trauma and chronic pain [26,27]. Multimodal interventions that integrate physical therapy, psychological counseling, and psychoeducation have demonstrated effectiveness in improving pain management and overall functioning [28]. Somatic and mind–body approaches, in particular, may complement traditional counseling interventions by directly targeting the physiological effects of trauma while simultaneously supporting emotional processing [28,29]. A growing body of evidence supports the effectiveness of interdisciplinary and multidisciplinary pain management programs, with studies demonstrating improvements in pain intensity, psychological functioning, and quality of life compared to usual care or single-modality interventions [30,31,32,33]. Despite growing support for interdisciplinary approaches, limited research has examined how such models function specifically within group counseling settings, where shared experiences, cohesion, and peer validation may further facilitate emotional and physical healing. Somatic and physiological regulation approaches that target breathing patterns, neuromuscular alignment, and autonomic balance may complement counseling interventions by addressing the embodied effects of trauma and chronic pain. Approaches such as Postural Restoration Institute (PRI)–informed interventions emphasize the integration of posture, respiration, and neuromuscular control to support physiological regulation and bodily awareness [34,35]. These mechanisms align with trauma research highlighting the role of autonomic regulation and embodied processing in recovery [35,36]. PRI principles provide a systems-oriented framework for understanding postural asymmetries, respiratory mechanics, and neuromuscular patterning. Originally developed by Ron Hruska [37], PRI conceptualizes the body as inherently asymmetrical, emphasizing the role of diaphragmatic function, lumbopelvic alignment, and patterned neuromuscular activity in shaping movement and pain experiences. Emerging evidence suggests that PRI-informed interventions may improve pain, range of motion, and functional movement patterns through targeting these underlying biomechanical and respiratory processes [38,39,40]. These principles are consistent with a broader body of rehabilitation research demonstrating that breathing mechanics, postural control, and neuromuscular coordination play a critical role in pain modulation and functional recovery [41,42]. From a biopsychosocial perspective, interventions targeting respiration and postural regulation may also influence autonomic nervous system functioning, linking physiological regulation with emotional and cognitive processes relevant to trauma and chronic pain.

The emphasis on respiration, postural alignment, and neuromuscular patterning within PRI also aligns with emerging research on the role of the autonomic nervous system in chronic pain and trauma-related conditions. Dysregulation of autonomic processes, particularly persistent sympathetic activation and reduced parasympathetic flexibility, has been implicated in both heightened pain sensitivity and the maintenance of posttraumatic stress symptoms [43,44]. Interventions that incorporate breathwork, somatic awareness, and physiological regulation may therefore serve as a critical bridge between physical and psychological domains of treatment, supporting integrated approaches to healing.

The present study addresses this gap by examining an interdisciplinary group intervention designed to concurrently target trauma symptoms and chronic pain. Guided by the biopsychosocial model, this study employed a convergent mixed-methods design to explore how participants’ experiences within an interdisciplinary group counseling intervention aligned with changes in trauma symptomology and pain levels [45].

### 1.2. Research Question

How do participants’ experiences with the interdisciplinary group intervention align with changes in their trauma symptomology and pain levels?

## 2. Materials and Methods

### 2.1. Research Design

To address the research question, we employed a convergent mixed-methods approach [46]. To evaluate trauma symptoms and levels of chronic pain, we utilized a quasi-experimental within-groups design. For qualitative analysis, we employed Thematic Analysis [47] to examine participant experiences.

### 2.2. Research Team and Trustworthiness

Qualitative analysis was conducted by three doctoral-level researchers. Reflexivity and bracketing were used to identify potential biases prior to and during coding [46,47,48,49]. In line with thematic analysis procedures [47], all team members familiarized themselves with the full dataset before and throughout the coding process. To enhance trustworthiness, the study incorporated multiple rigor strategies, including reflexive journaling to support researcher self-awareness, maintenance of an audit trail to document analytic decisions, and ongoing team debriefing to promote credibility and consistency in data interpretation.

### 2.3. Data Collection and Instrumentation

In the following sections, the quantitative and qualitative data collection and instrumentation for the mixed-methods pilot study are described.

#### 2.3.1. Quantitative Instrumentation and Data Collection

Following informed consent, participants completed demographic information and standardized measures at pre- and post-intervention. Trauma symptoms were assessed with the PCL-5 (α = 0.94) [50], fear of movement with the TSK (α ≈ 0.70–0.80) [51], and pain intensity and interference were tracked weekly using the Brief Pain Inventory (α > 0.85) [52]. Group cohesion was measured post-intervention using the Group Climate Questionnaire [53].

#### 2.3.2. Qualitative Instrumentation and Data Collection

Thematic Analysis [54] was the qualitative approach in this study to explore the research question. The data sources include weekly journal responses, weekly photographs, open-ended post-survey questions, and focus group interviews. The collection of photographs was influenced by the Photovoice methodology [55], which functioned as a primary element of the intervention. Central to Photovoice, participants captured photographs that they related to the challenges in their lives as well as the support received throughout the study, and they discussed these pictures within the group process [55]. Multiple data sources were used to enhance depth and triangulation: journals captured ongoing reflection, photographs facilitated embodied expression, surveys provided summative insight, and focus groups enabled collective meaning-making. This data collection method aligns with a trauma-informed approach, specifically as it relates to empowerment, agency, and choice for the participants [56,57].

### 2.4. Data Analysis

#### 2.4.1. Quantitative Data Analysis

Descriptive statistics were first computed to summarize the key variables and provide an overview of the data. Means, standard deviations, and ranges were calculated for each of the outcome measures. To explore potential group-level differences at baseline and post-intervention, independent samples t-tests were conducted for exploratory purposes only and were not intended to support inferential group comparisons. These t-tests compared the pre-intervention scores for each outcome measure across the three intervention groups to assess whether there were significant differences between the groups before the intervention began. Following the intervention, paired samples *t*-tests were conducted to examine within-group changes in trauma symptoms, fear of movement, and pain levels. These analyses were selected to provide preliminary insight into change over time within participants. This allowed for the assessment of within-group changes in trauma symptoms, fear of movement, and pain levels, offering insight into how each group responded to the intervention over time. Due to the repeated-measures structure of the data and small subgroup sizes, assumptions of independence and statistical power are limited; therefore, results should be interpreted cautiously as preliminary and descriptive rather than confirmatory. More advanced analytic approaches (e.g., linear mixed models) were not employed due to sample size constraints but are recommended for future research.

#### 2.4.2. Qualitative Data Analysis

Thematic analysis was employed to analyze qualitative data, following Braun and Clarke’s [47] six-phase approach: familiarization, initial coding, searching for themes, reviewing themes, defining and naming themes, and writing the report. This method allows for identifying patterns within the data, ensuring a rich, detailed account of participants’ experiences [58]. An inductive coding process was utilized, meaning themes emerged directly from the data rather than being predetermined by theoretical frameworks.

In alignment with reflexive thematic analysis [58], coding reliability was not established through statistical agreement; instead, a collaborative and reflexive coding process was used. The research team met regularly to compare codes, discuss interpretations, and refine the coding framework. Differences in coding were treated as opportunities for deeper analytic engagement, and discussions continued until a shared understanding of the data was reached.

Themes were refined iteratively through constant comparison, ensuring they captured the depth and complexity of participants’ narratives. Each team member generated codes on the journal entries and photograph data sources as the intervention was still in progress (starting at week 4). To minimize potential analytic bias, coding activities were conducted independently of intervention delivery. Researchers engaged in coding did not use emerging interpretations to inform or alter session content, facilitation style, or participant interactions. The intervention protocol was manualized and followed consistently across sessions, which helped ensure that ongoing analysis did not influence implementation. Additionally, the research team engaged in reflexive dialogue to remain aware of potential bias and to bracket emerging assumptions during both facilitation and analysis. After the intervention was completed (week 6), the open-ended post-survey responses were coded along with focus group transcripts. A team member was assigned to code one focus group transcript, while the other team members served to review and audit so that each member had a role in each stage. Furthermore, member checking occurred throughout the data collection phase (e.g., interviews), data analysis, and report writing [49]. Given the nature of the intervention itself, across the 6 weeks, participants were aware of the data they were contributing to and had the opportunity to revise or correct their responses. Throughout this process, the team maintained an audit trail documenting coding decisions and engaged in ongoing reflexive dialogue to examine how their perspectives may have shaped interpretation. Additionally, given the diverse data sources (i.e., journals, photographs, open-ended post-survey questions, focus group interviews), triangulation helped us validate the findings. After codes were generated, they were organized into categories [47] as preliminary themes. There were 12 preliminary themes. As a research team, these themes were discussed and grouped into 7 final themes. To enhance transparency in theme development, we outline the analytic progression from initial codes to final themes. Initial coding generated a range of codes reflecting participant experiences (e.g., bodily disconnection, avoidance behaviors, emotional distress, meaning-making, perceived benefits, and group relational processes). These codes were grouped into broader conceptual categories, which informed the development of 12 preliminary themes. Through an iterative process of team-based discussion and constant comparison, these preliminary themes were reviewed, refined, and grouped into 7 final themes. For example, codes related to bodily disconnection and avoidance behaviors were integrated into a broader theme reflecting the persistent burden of pain and trauma, while codes related to group safety, facilitator presence, and emotional expression were refined into a theme reflecting group-based emotional processing. The process of reviewing and grouping themes was guided by the following questions: (a) Do the themes make sense? (b) Does the data support the themes? (c) Are we trying to fit too much into a theme? (d) If themes overlap, are they really separate themes? (e) Are there themes within themes (subthemes)? [59]. These questions help prevent a common pitfall of using the main interview questions as the themes reflect the iterative and reflexive nature of thematic analysis [60].

#### 2.4.3. Intervention Design Summary

The interdisciplinary intervention integrated physical therapy and trauma-informed group counseling to address co-occurring chronic pain and trauma symptoms. The program was delivered across six weekly 90 min group sessions, with three groups receiving the same protocol. The outline can be seen in Table 1. Sessions were co-facilitated by a physical therapist and mental health counselor (with one group led by an alternate counselor and the same physical therapist).

Guided by the biopsychosocial model [26], each session incorporated biological, psychological, and social components. The biological component included gentle physical therapy exercises grounded in Postural Restoration Institute (PRI) principles, which focus on optimizing breathing mechanics, postural alignment, and neuromuscular coordination to facilitate autonomic regulation and reduce physiological stress responses [34,35]. These exercises were intentionally selected to complement trauma-informed counseling by targeting somatic dysregulation commonly observed in individuals with chronic pain and trauma histories [36,43].

Fidelity of the intervention was maintained through weekly self-reports completed by the facilitators. Fidelity was based on facilitator self-report, which may introduce bias due to lack of independent verification. The Interventionist Manual provided a structured outline for each session, detailing exercises, activities, prompts, and psychoeducation, all organized by time. Facilitators adhered to this manual to ensure that both the physical therapy and group counseling components were consistently implemented across sessions. The manual informed the “checklist” that interventionists completed each week. The overall fidelity rating across all sessions averaged 2.89 out of 3, indicating that the intervention was largely followed with slight variations.

It is important to note some deviations that occurred over the six-week intervention period. In Week 1, the mental health therapist was absent due to illness, requiring the physical therapist to conduct the session independently. A similar occurrence took place in Week 3, when the mental health therapist for Group 3 was absent due to sickness, and the physical therapist led the session alone. Facilitator absences may have influenced intervention consistency. Additionally, participant attendance varied across sessions: one participant in Group 1 was absent in Week 3, one participant in Group 2 missed Week 4, and one participant in Group 3 was absent in Week 5. Despite these absences, all participants continued to complete the weekly Brief Pain Inventory (BPI) assessments and journal prompts, ensuring continuity in data collection.

## 3. Results

### 3.1. Participants

Participants were recruited using purposive sampling [61] to ensure inclusion of individuals with lived experience of both chronic pain and trauma symptoms, which aligned with the study’s focus on examining this co-occurring population. Recruitment occurred through multiple channels, including flyers distributed in community and clinical settings, outreach to local clinics, and word-of-mouth referrals. These strategies were selected to reach individuals likely to meet inclusion criteria while reflecting real-world pathways through which individuals with chronic pain and trauma may access services. Participants met the following criteria: (a) age 19 years or older, (b) achieving a score of 31 or above on the Post-Traumatic Checklist-5 [50], (c) reporting chronic pain persisting for 3 months or more [62]. The reasoning for implementing this strategy is primarily for quality assurance [63]. Since the focus of this study is to gain an understanding of the experiences individuals have following their traumatic event, the PCL-5 served as a screener to ensure that participants appropriately matched that aim.

This study included 15 participants ranging in age from 25 to 81 years old (M = 52.47, SD = 18.66). The majority of participants identified as female (*n* = 13, 86.7%), while two participants identified as male (*n* = 2, 13.3%). All participants identified as White (100%). Participants were assigned to one of three groups, with Group 1 consisting of 3 participants, Group 2 with 6 participants, and Group 3 with 6 participants. The homogenous sample limits generalizability, particularly across racial and cultural contexts. All groups received the same interdisciplinary intervention, and participants self-selected into their groups based on availability. Participants described a wide range of chronic pain experiences, often involving multiple pain sites and overlapping medical conditions.

To comprehensively address our research question, the results section is structured to integrate both quantitative and qualitative findings. Results are organized into key categories, addressing where statistical outcomes align with participants’ lived experiences, as well as instances where subjective narratives provide deeper context to numerical trends. This mixed-methods approach allows for an interpretation of the data, where not only measurable changes in pain levels, trauma symptoms, and group cohesion can be reported but also illustrated through participant descriptions of how they engaged with and responded to the interdisciplinary intervention.

To integrate quantitative and qualitative findings, we employed a convergent mixed-methods integration approach [59,60], in which both datasets were analyzed independently and then merged during interpretation. Integration occurred through a process of joint interpretation, whereby quantitative outcomes (e.g., changes in trauma symptoms, pain, and fear of movement) were compared with qualitative themes to identify areas of convergence, divergence, and expansion.

Specifically, qualitative findings were used to contextualize and elaborate upon quantitative trends, providing insight into how participants experienced observed changes. In cases of convergence, qualitative data supported and enriched quantitative results; in cases of divergence, differences were examined to better understand variability in participant experiences. This integrative approach allowed for a more comprehensive understanding of the intervention’s impact across both measurable outcomes and lived experiences.

### 3.2. Trauma and Chronic Pain Experience

#### 3.2.1. Quantitative Results:

Pre-test assessments indicated substantial levels of trauma and fear of movement. The mean pre-test score for the Post-Traumatic Stress Disorder Checklist for DSM-5 (PCL-5) ranged from 36 to 56, with a mean score of 42.8 (SD = 5.71). This indicates that participants had trauma symptoms well above the clinical cutoff at baseline. Similarly, the pre-test scores for fear of movement, assessed using the Tampa Scale of Kinesiophobia (TSK), ranged from 22 to 37, with a mean score of 29.6 (SD = 4.70), reflecting moderate to high levels of fear of movement among participants at the start of the intervention. 

#### 3.2.2. Qualitative Findings:

Theme 1: The Persistent Burden of Pain and Trauma

Participants described chronic pain and trauma as relentless and ever-present, deeply influencing their ability to engage with daily life and contributing to feelings of hopelessness and isolation. They often framed their experiences as being “unrelenting,” with trauma and pain affecting their thoughts, emotions, and behaviors.

Subtheme 1.1: Disconnection from the Body

Participants reported this common experience of detachment from their bodies, marked by numbness, dissociation, and a lack of bodily awareness. Many described their actions and decisions as becoming automatic, with little conscious connection to their internal state. For example, Kina shared, “my body goes on auto-pilot; it just feels distant and numb,” reflecting the emotional and physical detachment many participants experienced. Similarly, Sarah stated, “Sometimes I notice nothing if I am distracting myself and other times I don’t notice anything except my pain,” showing how pain can overshadow other sensations and make emotional and physical awareness difficult. Janie reflected, “I just have to try to detach and that does make me more anxious but I also seem to have more energy in a way.” This dissociation, while providing temporary relief, often increased anxiety. Jay, too, explained, “Disconnecting is just part of what I always do. It is necessary for my job,” demonstrating how detachment became a necessary strategy for coping with the demands of work.

Subtheme 1.2: Avoidance of Life Activities

Participants shared their tendency to avoid activities or relationships that could trigger physical pain or emotional trauma. For instance, Jessica explained, “anything with arms above head is a huge trigger for my neck issues. This photo (raising a baby up in the air) shows something joyful & spontaneous that I shouldn’t do, but sometimes I do without thinking & pay for with increased pain for days.” Similarly, Kay shared her avoidance of activities like grocery shopping, saying, “This (photo) is my trunk full of groceries from Costco. I usually go with my adult son so he can lift and unload everything for me. I try to avoid taking groceries out of the car because it exacerbates my pain.” Others, like Emmie, acknowledged emotional vulnerability as a barrier to healing, stating that this photo “represents the struggle of allowing myself to cry and be vulnerable with people close to me. It’s something I generally ‘avoid’ and am working to overcome.”

Theme 2: Seeking Meaning and Understanding of Pain

Participants also wrestled with the meaning and purpose of their pain. While some framed their pain as a tool for growth or learning, others expressed frustration and confusion about its lasting nature. Kale captured the emotional struggle, saying, “Can you just lay off for awhile?” expressing the exhaustion of enduring chronic pain. Kay echoed this sentiment with, “When will it end?” signaling the frustration many participants feel about the seemingly endless nature of their pain. Similarly, Brynn questioned, “How can I get relief?” and Katy, grappling with her own limitations, inquired, “How do I decrease the pain?” However, Katrina reflected that, “I believe my pain wants me to share what I have learned. And I have been able to help others as a result of walking through my own pain. The only way out is through.” These reflective moments highlight the search for meaning that often accompanies an understanding of how to manage their suffering.

### 3.3. Feasibility and Acceptability

#### 3.3.1. Quantitative Results

The feasibility and acceptability of the intervention were assessed through several measures, including adherence, drop-out rates, and session fidelity. The overall attendance rate was 0.9667, with three participants missing one session each. Importantly, no participants dropped out of the study, indicating a high level of engagement and commitment to completing the intervention. This suggests that the intervention was well-received and feasible for participants to attend. In addition to attendance and retention, acceptability of the intervention was reflected in participants’ qualitative feedback and behavioral engagement. Participants consistently described the intervention as beneficial and expressed a desire for continued participation, with several indicating they would recommend the group to others or be willing to engage in future iterations. These responses suggest a high level of perceived value and acceptability of the intervention experience.

To assess adherence, we also tracked session fidelity. Intervention fidelity was assessed using a structured checklist completed by facilitators at the end of each session. Each item on the checklist corresponded to a core component of the intervention (e.g., physical therapy exercises, psychoeducation, and group processing elements). Items were rated using a 3-point scale: 0 = not met, 1 = partially met, and 2 = fully met. For each session, item-level scores were averaged to generate a session-level fidelity score. These session-level scores were then averaged across all sessions to produce an overall fidelity rating, with higher scores indicating greater adherence to the intervention protocol. The mean fidelity score across sessions was 2.89 out of 3, suggesting that the intervention was implemented with high consistency and adherence. Fidelity ratings were based on facilitator self-report, which may introduce bias due to the absence of independent observation.

#### 3.3.2. Qualitative Findings

Theme 3: Structure and Group Size

The intervention’s feasibility and acceptability were shaped primarily by feedback on its structural design and integration of physical therapy with mental health components. Group length, session structure, and the integration of physical therapy and mental health components influenced the overall participant experience. Participants appreciated the balance between physical therapy exercises and the group counseling aspects of the program. Some felt that the structured session flow enhanced their focus and emotional engagement. Others provided helpful suggestions for future improvement of the intervention. Janie expressed satisfaction with the duration and balance of the group sessions, stating, “I liked the time, having the hour and a half was like just the right amount of time to do the PT and talk about things. It was a good way to balance it out and nothing felt rushed.” Jay similarly highlighted the value of doing physical therapy at the beginning of each session in preparing participants for the emotional work in the group, noting, “for some reason today, like it woke me up… it kind of puts me in a different mind space.” Emmie shared a similar sentiment stating, “I was far more relaxed after doing the PT. And I think that helped me even focus or pay attention better when we did the group activities.” Robert also found the combination of physical therapy and group discussion to be helpful, stating, “I think just when you see that almost instant result with the PT part of it, being locked up and then going to neutral, I think that just does something internally to you.”

Additionally, some participants, such as Kay, suggested limiting group size, “I thought the group size (6 members + 2 facilitators) was a little big for sharing. I think 4 group members is an ideal size.” Hannah echoed this same sentiment by stating, “I agree with a smaller group size. The only other thing I would add is having a short PT exercise at the very end before we leave,” acknowledging it would be nice to start and finish with the physical therapy component. These suggestions provide small practical changes that can further improve future participant experience with this intervention.

Theme 4: Sustainability and Accessibility

Several participants expressed interest in the long-term sustainability and potential expansion of the intervention. Many expressed a desire to continue participating in the group or expand it to a broader population. Robert suggested extending the group length from six to eight weeks, stating, “either make the group longer. Perhaps 8-weeks instead of 6-weeks. Or create a 2.0 group.” Kale shared a similar desire for continued engagement, saying, “I wish I would have a couple more sessions to really understand not only more, but to continue the connection with these people.” Similarly, Brynn added, “Yes, I highly recommend this, and I also have two friends wanting to do it.” Janie expressed that she wishes this could be more available in other places, “this really needs to be passed on even in hospitals and clinics.” This willingness to continue the intervention reflects a certain level of perceived value in addressing both chronic pain and trauma in this format.

### 3.4. Emotional and Physical Outcomes

#### 3.4.1. Quantitative Results

**Figure 1:**
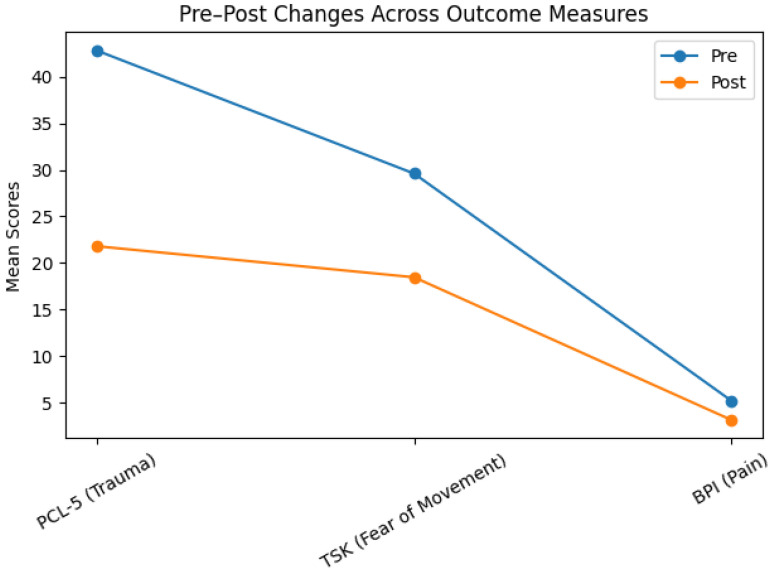
Pre-post changes across outcome measures.

Noticeable improvements were observed in the quantitative measures related to trauma symptoms, fear of movement, and pain levels. Please see Figure 1. Specifically, trauma symptoms, as measured by the PCL-5, showed a significant decrease from pre-test (M = 42.8, SD = 5.71) to post-test (M = 21.8, SD = 10.12), *t*(14) = 9.42, *p* < 0.001, *d* = 2.43, indicating a large effect size. Fear of movement, assessed using the Tampa Scale of Kinesiophobia (TSK), also demonstrated a significant reduction from pre-test (M = 29.6, SD = 4.70) to post-test (M = 18.47, SD = 4.55), *t*(14) = 11.47, *p* < 0.001, *d* = 2.96, reflecting a large effect size. Pain levels, measured by the Brief Pain Inventory (BPI), decreased from pre-test (M = 5.22, SD = 1.54) to post-test (M = 3.16, SD = 1.30), *t*(14) = 5.94, *p* < 0.001, *d* = 1.53, indicating a large effect size. Effect sizes should be interpreted cautiously given the small sample size and within-group design.

#### 3.4.2. Qualitative Findings:

Theme 5: Physical and Emotional Benefits

The intervention was overall well-received by participants, with many reporting meaningful benefits in pain management, emotional regulation, and well-being. This theme captures the extent to which the intervention produced observable improvements. Some of which include immediate pain relief, while others were related to more sustained benefits such as improved sleep, relaxation, and reduced chronic symptoms. Presley described the immediate relief she experienced from physical therapy exercises, noting, “It’s amazing. [the exercises] unlocked me instantly. I mean instantly, and then the pain dropped to zero.” She later described that, “I felt very exhausted after I left each session, but I will say the fruit, what I got out of this, was so much more. It is more than I’ve ever experienced in just physical therapy.” In a similar light, Emmie shared how she appreciated this format within a group setting, particularly stating that, “I have been able to accomplish more in these sessions that I do in my individual therapy sessions.”

Others also described the emotional growth they experienced. Jessica expressed gratitude for the emotional healing she experienced, stating, “I thought it was legitimately beneficial, and caused me to look at some things and finish dealing with trauma that I haven’t gone back to in many years. Participating in the group brought some things to the surface that I don’t think had been completely dealt with.” The combination of physical therapy and emotional processing provided observable shifts for experiences outside of the sessions as well. Hannah shared with the group, “I have never slept better than I have in years. The best I have slept in 20 years started because of this group.” In a similar way, Robert, reflecting on his long-term symptoms, shared a significant shift he experienced, “this is the first week (week 6) in 15 years that I have not gotten sick from eating. And it’s not because I didn’t try eating, I did. But this just typically after a meal I just started vomiting. And this was the first week in over a decade that that did not happen. I credit this group 100%.” This participant’s statement provides an example of how the intervention contributed to well-being.

Theme 6: Increased Mind–Body Awareness and Emotional Healing

Participants gained a greater understanding of the connection between their emotions and physical well-being. Janie remarked, “It like suddenly releases your nervous system and I feel so loose and I feel like that does affect the emotional… physically opening your body up, unlocks and can like make your emotions more open.” This newfound awareness of the mind–body connection facilitated emotional healing and allowed participants to process trauma more effectively. For others, the realization of the interconnectedness between their body and emotions was equally impactful. Hannah remarked, “I never knew that there’d be so much interconnection with trauma and the physical body,” emphasizing how the intervention allowed her to recognize the link between her emotional trauma and her physical symptoms. This realization led to a deeper understanding of how addressing one’s emotional state could alleviate physical pain, and vice versa.

Participants also noted the practical benefits of learning mind–body regulation techniques during the intervention. Brynn stated, “Personally, like my takeaway to take home and to implement the PT exercises that when I would do those things that it would make me emotional. I had never experienced something like that before.” This emotional release during physical therapy exercises revealed to her how trauma was stored in the body and how movement could release it, allowing for emotional processing. For many, the ability to regulate emotions through mind–body techniques became an important coping skill. Susie noted, “I took away the exercises for the vagus nerve a lot at my workplace. Sometimes I had to repeat over and over again. But it helped me tremendously there.” This comment highlights the practical application of the emotional regulation strategies, not only during sessions but also in participants’ daily lives. Learning how to manage their emotional responses to stress through physical exercises offered tangible improvements outside of the intervention. Brynn further reflected, “It’s something I’ve never experienced before, the emotional release I feel through physical activity. When I do the PT exercises, it’s like a whole new way of connecting to myself that I didn’t know was even possible.” This statement underscores how participants were able to access a new level of self-awareness and emotional healing through the combination of physical therapy and counseling.

### 3.5. Group Cohesion and Support

#### 3.5.1. Quantitative Findings

The mean group cohesion score was 62.4 (SD = 4.52) as measured by the Group Climate Questionnaire [53]. This score reflects the general level of connection and mutual support experienced by participants during the intervention. Higher group cohesion scores suggest that participants felt a strong sense of connection with others in the group. Group-level analyses were conducted to compare the pre- and post-test scores for each of the three groups. For trauma symptoms (PCL-5), Group 1 showed a trend toward significance, with a decrease from pre-test to post-test, *t*(4) = 3.94, *p* = 0.059. Both Group 2 (*t*(4) = 6.42, *p* = 0.0014) and Group 3 (*t*(4) = 5.14, *p* = 0.0036) demonstrated significant decreases in trauma symptoms, reflecting improvement in PTSD symptoms following the intervention.

For fear of movement (TSK), all three groups showed significant reductions. Group 1 had a significant decrease, *t*(4) = 6.43, *p* = 0.0234, with Group 2 showing a more pronounced reduction, *t*(4) = 8.43, *p* < 0.001, and Group 3 also reporting a significant decrease, *t*(4) = 6.18, *p* = 0.0016. These results suggest that participants across all groups experienced reduced fear of movement as a result of the intervention. Pain levels were similarly reduced across all groups, with Group 1 showing a significant decrease, *t*(4) = 6.98, *p* = 0.0199, Group 2 reporting a significant decrease, *t*(4) = 4.50, *p* = 0.0064, and Group 3 showing a significant decrease, *t*(4) = 3.05, *p* = 0.0284. These findings suggest that participants experienced a reduction in pain levels after participating in the intervention. Given the small group sizes, these group-level analyses should be interpreted with caution, as estimates may be unstable and not generalizable. Additionally, the use of multiple t-tests increases the risk of Type I error, and findings should therefore be considered exploratory.

#### 3.5.2. Qualitative Findings

Theme 7: Deepened Emotional Processing Through Group Work

The group setting provided a safe and supportive space for emotional expression, which was initially challenging but ultimately facilitated deep emotional processing. As Kale reflected, “When we’re talking about our pain journeys… what helped me was hearing other people’s stories… I’m not the only person that thinks like that.” Kay shared something similar in that, “I’m not the only one that has those thoughts. Almost verbatim, you know, somebody else was thinking the exact thing I have though regarding their pain. You can start feeling really isolated like, oh, I’m the only one that feels like this or I’m the only one that this has happened to. So, it’s very helpful for me to be like, oh my gosh, I’m actually pretty normal.” This sense of connection and validation within the group played a crucial role in participants’ emotional growth. Kale continued, “I can remember the first time that you got teary-eyed in the group. And I didn’t know all of that but somehow I knew that that was a big deal for you. It’s like I could feel the healing happening for you in that moment.” Katy echoed this sentiment, noting, “I feel like I’ve learned so much from this group that they’re just wonderful.” Over time, participants grew more comfortable sharing their vulnerabilities, as Emmie shared, “I was able to experience hard things in front of people without feeling shame behind that. The validation part too. It was comforting but feels kind of awkward. At the same time like it is hard to receive it but it was also so good. It started getting a little easier to receive.” The group’s supportive and validating environment not only alleviated feelings of isolation but also fostered a deeper sense of connection, helping participants process their emotional experiences with greater ease and acceptance over time.

Subtheme 7.1 Engaged and Empathic Group Facilitators

This deepened emotional processing was supported by the group facilitators, who helped to create a safe, nurturing space for participants to explore their trauma. Jessica shared initial apprehension and how that changed, stating, “prior to joining, thinking about working through trauma was intimidating. I’d never been in a situation where I had to share in a group about trauma much less I’ve never really shared about it in general… the paperwork, it said I would never be forced to share so that’s why I showed up. But [therapist) did such an incredible job of creating a safe space and I saw what these brave women sharing and I was identifying with them. And before I knew it, all this came out of me and I feel so much more free.” Kina added to this by describing, “There were moments when the moments got deep, and you [therapist] allowed it to be deep and you manage this Herculean task. You always made sure that everyone shared, but everyone shared just the right amount for them.” Jay discussed, “at least from my experience, when you get into some of these environments talking about feelings and hurts and traumas, it can get out of control, sometimes it can get awkward, it can get, you know, you can go down some weird (crap) where you can go down some roads or paths where you shouldn’t probably be going down but (therapist) had an ability to reel us back in and they did a really good job of like validating and keeping things flowing.” Robert added, “There was just such a care from both of them to help us not only get better, but to address, whatever the trauma is and there’s truly legitimate compassion and care.” The facilitators’ ability to create a safe, supportive, and compassionate environment was instrumental in allowing participants to engage deeply in their healing process, fostering emotional growth and connection throughout the group.

### 3.6. Physical Therapy Outcomes

In addition to self-reported measures, physical therapy assessments of postural neutrality were included as an outcome measure. Postural neutrality refers to the degree of balanced alignment and symmetry across the body, particularly in relation to respiratory function, pelvic positioning, and neuromuscular coordination, as conceptualized within Postural Restoration Institute (PRI) frameworks [34,35].

Neutrality was assessed by the physical therapist using standardized PRI-informed clinical observations, which included evaluation of postural alignment, breathing patterns, and functional movement indicators (e.g., symmetry in weight-bearing, rib cage positioning, and pelvic alignment). Scores were assigned based on the extent to which participants demonstrated balanced and regulated postural positioning following intervention activities. While PRI-based assessments are used in clinical practice to evaluate postural and respiratory function, formal psychometric validation of these specific neutrality scoring procedures remains limited. As such, this measure should be interpreted as a clinically informed indicator of postural change rather than a standardized or norm-referenced assessment. Neutrality scores showed significant improvement over time, *t*(14) = 3.94, *p* = 0.001, 95% CI [0.42, 1.43], Cohen’s *d* = 1.02, suggesting that participants experienced greater physical stability and reduced asymmetry following the intervention.

## 4. Discussion

The current study examined the effectiveness of an interdisciplinary intervention designed to address co-occurring chronic pain and trauma. Findings from both qualitative and quantitative analyses suggest that integrating physical therapy with trauma-informed group counseling may provide meaningful benefits for individuals experiencing these complex and interconnected conditions. Quantitative results indicated reductions in trauma symptoms, fear of movement, and pain levels, while qualitative findings illuminated the emotional, psychological, and somatic processes underlying these changes.

### 4.1. Trauma and Pain Relief

Participants entered the intervention with elevated trauma symptoms and fear-avoidance behaviors, reflecting the intertwined nature of chronic pain and trauma-related distress. Following the intervention, both trauma symptoms and fear of movement demonstrated substantial reductions, suggesting that addressing psychological trauma and somatic experiences concurrently may disrupt maladaptive fear-avoidance cycles. These findings support theoretical models proposing a bidirectional relationship between trauma-related distress and pain perception, in which reductions in emotional dysregulation may contribute to decreased fear of movement and improved functional engagement.

Although pain intensity also decreased, weekly variability suggests that short-term fluctuations in chronic pain remained present. Such variability is consistent with literature indicating that pain experiences often fluctuate daily and may require longer intervention durations to produce stable, long-term changes [64,65]. Thus, while trauma symptoms and fear-avoidance behaviors appeared highly responsive to the intervention, sustained pain reduction may depend on longer or more individualized treatment approaches.

These findings can also be understood through the fear-avoidance model of chronic pain, which posits that individuals who perceive pain as threatening may develop avoidance behaviors that contribute to the maintenance of pain and disability [66]. The observed reductions in fear of movement suggest that participants may have begun to disrupt this cycle by engaging in safe, supported movement experiences and increasing tolerance for bodily sensations.

Additionally, from a gate control theory of pain perspective, emotional and cognitive processes play a central role in modulating pain perception [67]. Improvements in trauma symptoms and emotional regulation observed in this study may have influenced pain experiences by altering central nervous system processing. Together, these findings support the bidirectional relationship between psychological and physiological processes and highlight the value of interventions that simultaneously target both domains.

### 4.2. Mind–Body Awareness

The physical therapy component of the intervention was grounded in Postural Restoration Institute (PRI) principles, which emphasize breathing mechanics, neuromuscular alignment, and postural balance to support autonomic regulation and embodied awareness [34,35]. From a trauma-informed perspective, these PRI-informed somatic regulation exercises may help reduce physiological threat activation and increase bodily stability, thereby creating a foundation for emotional processing. These findings can be further understood through the lens of somatic experiencing [36], which posits that trauma is stored within the body and that restoration of autonomic regulation through bodily awareness and movement facilitates emotional processing and integration. Participants’ reported increases in mind–body awareness and emotional release are consistent with this framework, suggesting that engaging physiological systems directly may support access to and processing of trauma-related experiences.

Additionally, these results align with the biopsychosocial model [26], which conceptualizes chronic pain and trauma as interconnected across biological, psychological, and social domains. The integration of physical therapy and group counseling in the present study reflects this multidimensional approach, addressing physiological regulation (biological), emotional processing (psychological), and relational support within the group context (social). Together, these findings support the value of interdisciplinary interventions that target multiple levels of functioning to promote holistic healing.

### 4.3. Group Support and Emotional Regulation

The group counseling context emerged as a critical mechanism of change. Participants described feeling validated, understood, and less isolated, highlighting the therapeutic value of shared experiences in addressing both trauma and chronic pain. Greater group cohesion was associated with larger reductions in trauma symptoms, suggesting that relational safety and peer validation may facilitate emotional processing and resilience. This finding aligns with established literature on the therapeutic factors of group counseling, particularly universality, cohesion, and interpersonal learning [53,68].

Facilitator presence and structure further supported emotional regulation and engagement in the intervention. Participants noted that consistent guidance and validation created a safe environment in which they could explore trauma-related experiences while remaining grounded in the present moment. This underscores the importance of skilled facilitation in interdisciplinary group interventions, particularly when integrating somatic and emotionally evocative processes.

These findings align with Yalom’s therapeutic factors in group psychotherapy, particularly universality, group cohesion, and interpersonal learning [68]. Experiences of validation and recognition that others shared similar struggles reflect the therapeutic factor of universality, which can reduce feelings of isolation and normalize distress. The development of trust and emotional safety within the group suggests strong group cohesion, which has been consistently linked to positive treatment outcomes.

Furthermore, opportunities for participants to share experiences and receive feedback from others may have facilitated interpersonal learning, supporting both emotional insight and behavioral change. These relational processes likely contributed to participants’ ability to engage more deeply in both emotional and somatic aspects of the intervention, reinforcing the importance of group-based approaches in addressing complex, co-occurring conditions such as trauma and chronic pain.

While findings from the present study suggest meaningful improvements in trauma symptoms, fear of movement, and pain, these results should be interpreted within the context of a small, non-controlled pilot design. As such, conclusions regarding intervention effectiveness remain preliminary. Rather than demonstrating causal effects, the findings provide initial evidence of potential benefit, highlighting the promise of interdisciplinary, mind–body interventions for individuals experiencing co-occurring trauma and chronic pain. Future research employing larger samples and controlled designs is needed to more rigorously evaluate intervention efficacy.

## 5. Limitations

Several limitations should be considered when interpreting the findings. First, the small sample size limits generalizability, particularly to more diverse populations and individuals with varying trauma histories [69]. Second, although reflexivity and bracketing procedures were employed, researcher bias may still have influenced qualitative interpretation given the subjective nature of thematic analysis [70]. Third, participants received a $100 incentive, which may have influenced motivation or retention despite reports of minimal perceived impact [71]. Finally, reliance on self-report measures introduces the possibility of response biases, including social desirability and overestimation of treatment effects [72,73]. Additional methodological limitations should be considered. First, participants were recruited using purposive, non-random sampling, which may introduce selection bias and limit the generalizability of findings beyond individuals who were motivated to participate in an interdisciplinary intervention. Second, the small sample size and within-group design limit statistical power and increase the likelihood of unstable estimates. The use of multiple t-tests further introduces the potential for Type I error, and group-level analyses with very small subgroup sizes should be interpreted cautiously. Collectively, these statistical considerations reinforce that findings should be viewed as preliminary and exploratory rather than confirmatory.

## 6. Future Research

Future research should further evaluate the effectiveness of interdisciplinary trauma-informed group interventions for chronic pain through more rigorous designs. Studies incorporating comparison groups and randomized controlled trials would strengthen causal inferences regarding the intervention’s impact relative to standard treatments. Longitudinal research is also needed to examine the durability of treatment effects on both trauma symptoms and pain outcomes. Additionally, the use of advanced analytic approaches (e.g., linear mixed models) may better account for repeated measures and individual variability across time.

Future studies should also explore potential moderators of treatment response, including demographic and clinical characteristics, to identify which populations benefit most from interdisciplinary approaches. Finally, research examining specific intervention components and the feasibility of telehealth delivery may inform optimization and scalability of interdisciplinary models for individuals experiencing co-occurring trauma and chronic pain.

## 7. Conclusions

The findings of this mixed-methods pilot study provide preliminary support for the feasibility, acceptability, and potential clinical utility of an interdisciplinary group intervention integrating trauma-informed counseling with physical therapy grounded in PRI principles. High attendance, zero attrition, and strong fidelity underscore the practicality of this model, while reductions in trauma symptoms, fear of movement, and pain suggest meaningful change across both psychological and physiological domains. However, these findings are best understood as preliminary rather than confirmatory, given the small, non-controlled design. Within this context, the study contributes to a growing body of literature positioning interdisciplinary, mind–body interventions as a promising direction for addressing complex, co-occurring conditions such as trauma and chronic pain. Clinically, the integration of somatic regulation strategies within group counseling, alongside collaboration between mental health professionals and physical therapists, represents a meaningful expansion of trauma-informed care that aligns with biopsychosocial and embodied frameworks. Continued research with larger, more diverse samples and rigorous methodologies is needed to evaluate the durability, mechanisms, and scalability of this approach. Nonetheless, this study offers an important step toward bridging physical and mental healthcare, emphasizing the need for holistic, integrative models capable of addressing the multifaceted nature of human need and distress.

## Figures and Tables

**Table 1:** Overview of the Interdisciplinary Intervention Structure Across Six Sessions.

Session	Focus	Components
Week 1	Orientation and Awareness	Introduction to group; overview of trauma and chronic pain; foundational breathing and postural exercises; establishment of group norms
Week 2	Mind–Body Connection	Psychoeducation on the nervous system; continued breathing and postural exercises; introduction to mind–body awareness; group reflection
Week 3	Emotional Awareness	Identification of emotional responses; exploration of connections between emotional and physical experiences; introduction of regulation strategies; group discussion
Week 4	Regulation and Integration	Application of regulation techniques; integration of physical and emotional awareness; deeper group processing
Week 5	Interpersonal Processing	Exploration of shared experiences; development of group cohesion; examination of relational dynamics; continued applied exercises
Week 6	Consolidation and Reflection	Review and integration of key concepts; reflection on participant experiences; discussion of continued application beyond the intervention

## Data Availability

The original contributions presented in this study are included in the article. Further inquiries can be directed to the corresponding author.

## Abbreviations

The following abbreviations are used in this manuscript:

PRI	Postural Restoration Institute
PTSD	Posttraumatic Stress Disorder
